# Epidemiology, classification and treatment of olecranon fractures in adults: an observational study on 2462 fractures from the Swedish Fracture Register

**DOI:** 10.1007/s00068-021-01765-2

**Published:** 2021-08-03

**Authors:** Anders Brüggemann, Sebastian Mukka, Olof Wolf

**Affiliations:** 1 Department of Surgical Sciences, Orthopaedics, Uppsala University, Uppsala, Sweden; 2 Department of Surgical and Perioperative Sciences, Orthopaedics, Umeå University, Umeå, Sweden

**Keywords:** Olecranon fracture, Epidemiology, Swedish Fracture Register, Treatment

## Abstract

**Purpose:**

This nationwide study aims to describe the epidemiology, fracture classification and current treatment regimens of olecranon fractures in adults.

**Methods:**

We performed a descriptive study based on registered data from the Swedish Fracture Register (SFR). All non-pathological olecranon fractures reported between 1 January 2014 and 31 December 2018 in patients aged ≥ 18 years were included. Data on age, sex, injury mechanism, fracture classification (according to the modified Mayo classification system), primary treatment and seasonal variation were analyzed. We compared patients < 65 with those > 65 years regarding injury mechanism, distribution of fracture types and subsequent treatment.

**Results:**

In total, 2462 olecranon fractures were identified in the SFR. Median age was 66 years and 65% were women. Of all fractures, 303 (12%) were proximal avulsion, 1044 (42%) simple central, 717 (29%) comminuted central and 398 (16%) distal olecranon fractures. Nonoperative treatment was performed in 21% of the patients < 65 and 35% of the patients > 65 years. Tension band wiring was used for most simple central fractures. Plate fixation was used in almost half of the operatively treated fractures classified as unstable comminuted central and distal olecranon fractures. Men show a higher proportion of high-energy trauma than women in both age groups.

**Conclusion:**

Isolated fractures of the olecranon occur after a low-energy trauma, especially in older women (> 65 years). Non-operative treatment is common in uncomplicated fractures and operative treatment in more complex fractures nationwide. A shift to plate fixation in the more unstable fracture patterns is observed. These results may help health care providers and clinicians gain a better understanding of isolated olecranon fractures.

## Introduction

Olecranon fractures can be either isolated fractures of the extensor mechanism in the elbow or of more complex nature, including fracture dislocations. These fractures occur in all age groups but may be an early osteoporotic fracture given the higher incidence in elderly patients [1]. The injury mechanism is predominately a simple fall and open fractures are rare [1].

The size of the fractured olecranon tip decides the stability of the ulnohumeral joint, dictating the classification of the fracture and thus treatment. The Mayo classification is the most commonly used classification system dividing the fractures into type I–III, representing undisplaced, displaced and distally displaced with volar ulnar displacement. The type I–III fractures are further divided into A (non-comminuted) and B (comminuted) fractures [2].

The treatment of olecranon fractures ranges from non-operative to operative treatment with sutures, tension band wiring (TBW), screw or plate fixation depending on patient factors, fracture configuration and surgeon preference [3–6].

Copious reports have been conducted on treatment regimens and smaller randomized trials. However, only one small single-center report describes the epidemiology of olecranon fractures [1].

Therefore, our study aimed to describe the epidemiology of olecranon fractures including injury mechanism, fracture classification, sex and age distribution, primary treatment and seasonal variation using the national Swedish Fracture Register (SFR). By this approach, we aimed to investigate whether there are differences between men and women or younger and older patients regarding injury mechanisms, fracture pattern and subsequent treatment.

## Materials and methods

### Study design and setting

This observational cohort study was designed based on data derived from the SFR.

The SFR, established in 2011, is a national quality register for the management of fractures and treatment. Detailed data on patient and fracture characteristics, injury mechanism and fracture treatment are registered prospectively at each affiliated department via a pre-specified digital form by the treating physician. Only patients with a permanent Swedish personal identity number and fractures that have occurred in Sweden are registered. In the SFR, fractures are mainly classified according to the AO/OTA classification system. Several studies have found the registration in the SFR to have high accuracy and validity [7–9]. The proportion of departments affiliated with the SFR has increased gradually, and at the start of this study (January 2014), 40% of affiliated departments were active. By the end of the study (December 2018), participation had risen to 85%, with a coverage of more than 80% of the Swedish population. More than 330,000 fractures had been registered in the SFR by the end of 2018.

**Fig. 1:**
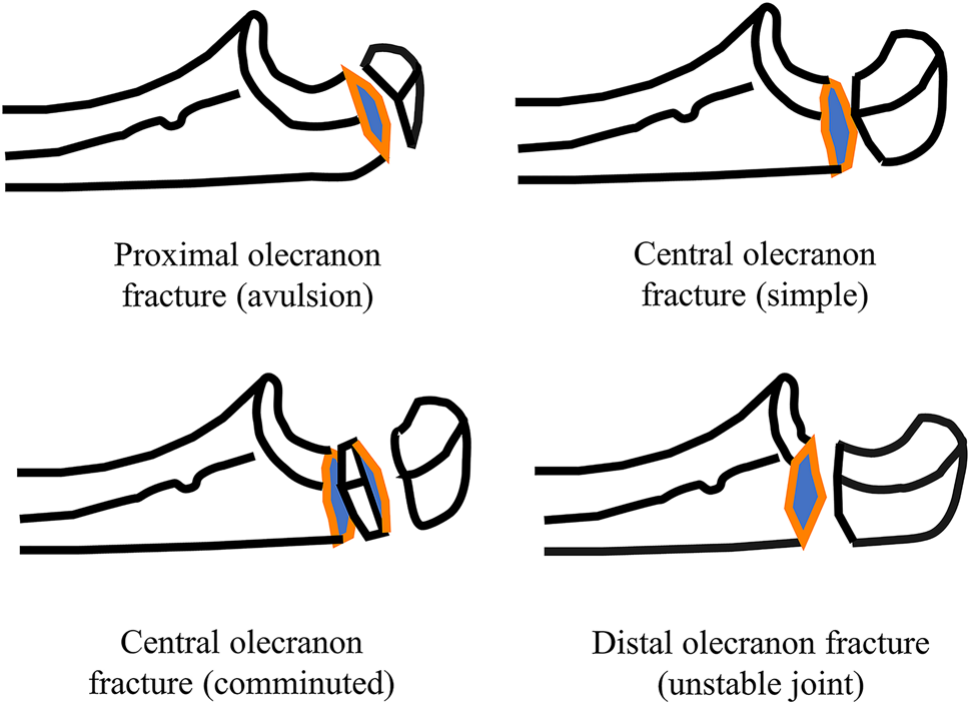
Schematic description of the four fracture subtypes included in this study

The registration of proximal forearm fractures in the SFR includes fracture of the proximal radius and/or proximal ulna, accompanied dislocation and whether the fracture was open. First, the potential fracture of the proximal radius is registered, then the proximal ulna, followed by a question about whether there is an accompanied dislocation or not. Periprosthetic fractures can also be classified separately. Groups of fractures can be distinguished with either isolated proximal radius or ulna fractures or complex fracture configurations. In addition, information on open fractures as described by the Gustilo–Anderson (G–A) classification is available. The olecranon fractures are classified in the SFR according to a modified Mayo classification into four groups: proximal avulsion (Mayo types I A–B), simple central (Mayo type II A), comminuted central (Mayo type II B) and distal olecranon fracture (Mayo types III A–B, Fig. 1). Treatment is registered with the chosen type of treatment (non-operative or operative). Operative treatment is further specified into TBW, screw fixation, plate fixation and combined method.

### Patient selection

All non-pathological fractures of the olecranon (ICD code S52.00/S52.01 (closed/open)) registered in the SFR between 1 January 2014 and 31 December 2018 in patients aged ≥ 18 years were included. We excluded all isolated fractures of the proximal radius, fractures of the coronoid and proximal ulnar shaft and combinations of more than one fracture, including fracture dislocations.

### Outcome variables

Data on age, sex injury date, mechanism and type (high- or low-energy trauma), fracture classification (type, side, open/closed fracture) and treatment were analyzed.

The cause of injury was categorized as a simple fall, a fall from a height, an unspecified fall, a transportation accident or any other cause. Transportation accidents were grouped into bicycle, motorbike or other, and fracture types were analyzed according to the modified Mayo classification. Primary treatment was studied in the following groups: TBW, screw fixation, plate fixation or a combined method.

### Statistics

Nominal variables are presented as proportions of all registered fractures and scale variables as means ± standard deviation (± SD) if normally distributed, and as median with inter-quartile range (IQR) for non-normaly distributed data. All statistics, including mean values, percentages, tables and figures, were calculated using the R version 4.0.3 software package[10].

### Ethics

This study was approved by the Regional Ethical Committee in Uppsala (Dnr: 2015/509 and 2019/011006).

## Results

**Fig. 2:**
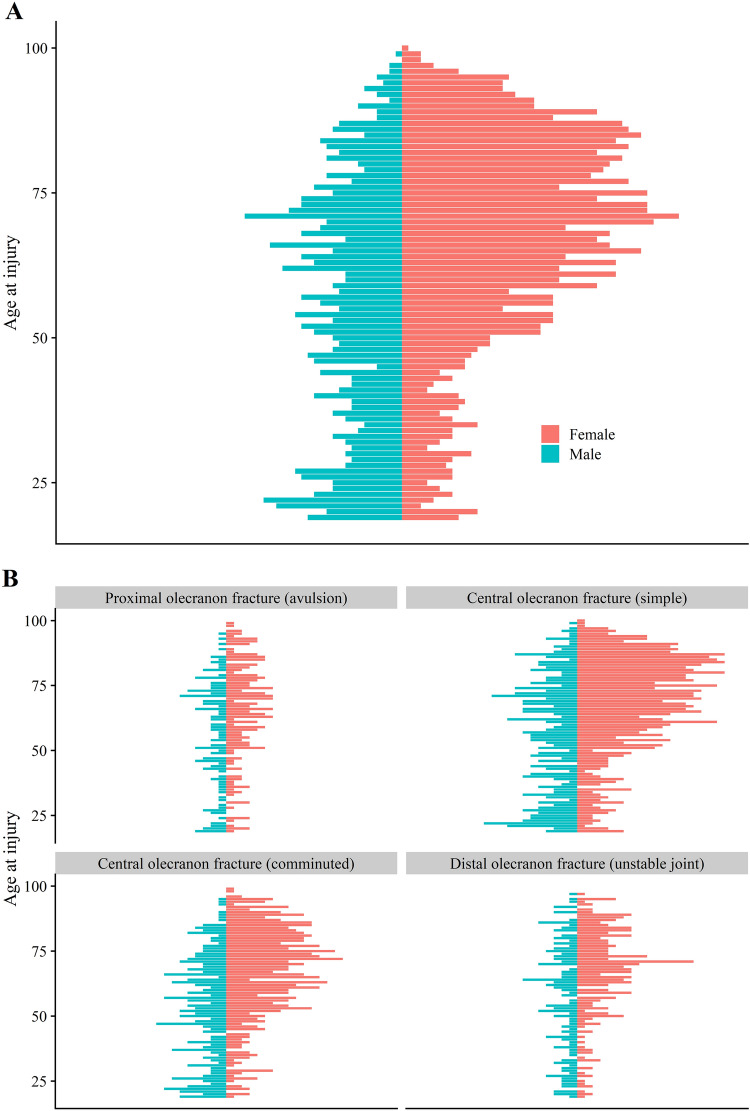
Distribution of age at injury for **A** all fractures and **B** the four respective subclassifications

**Fig. 3:**
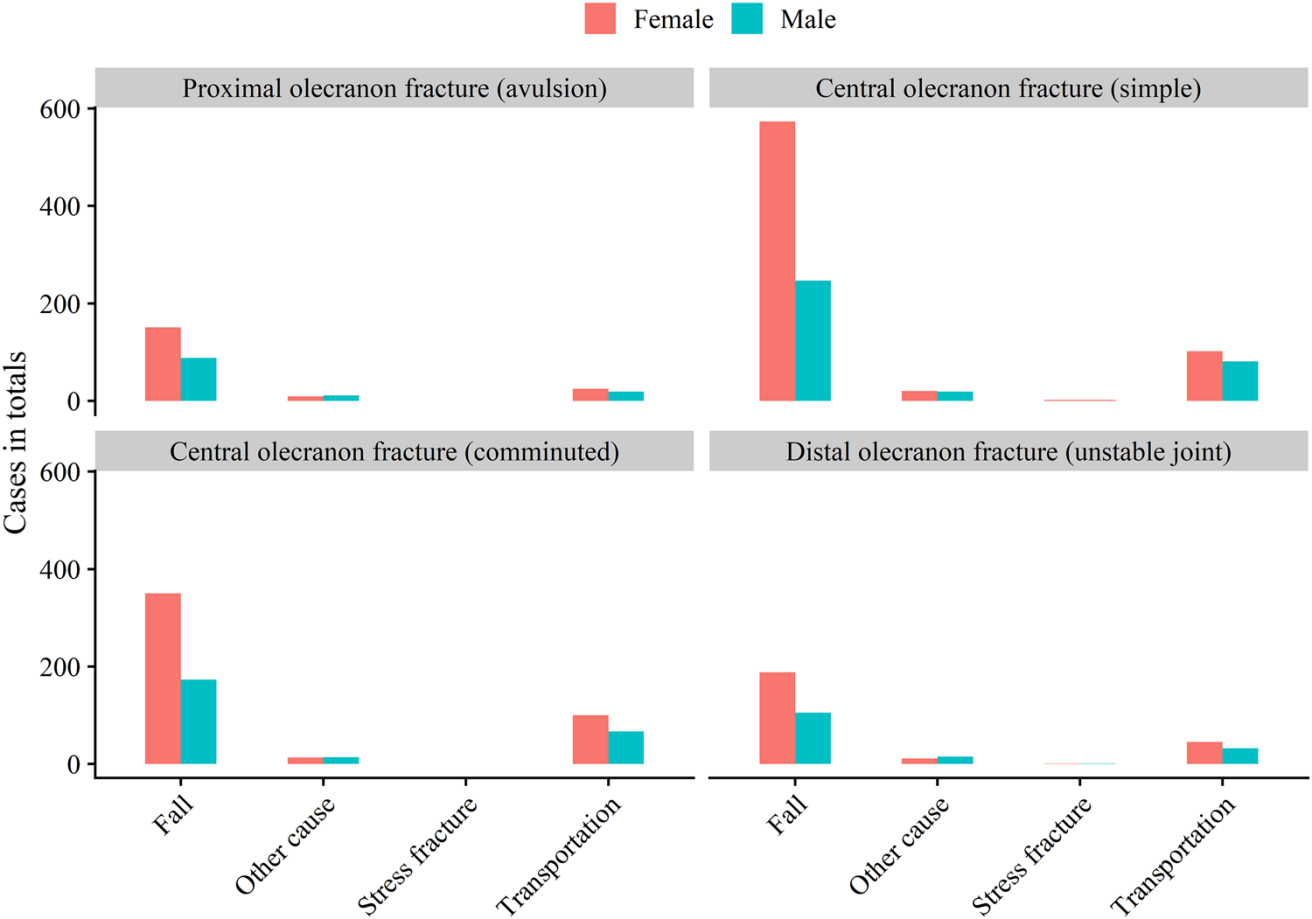
Distribution of the injury mechanism for the four fracture subgroups

In total, 3233 proximal forearm fractures were extracted from the SFR (ICD-10 code S 52.00/52.01). After exclusion, 2462 olecranon fractures were included in this study. Twelve patients sustained simultaneous bilateral fractures and 19 patients experienced a re-fracture on the same side during the study period. Of all fractures, 65% occurred in women and the median age was 66 (IQR 50–79) years. Women were older (median age 70 years, IQR 57–81) than men (56 years, IQR 37–72). Only small differences were found between the four subtypes in demographics, injury mechanism and high- or low-energy trauma (Table 1; Fig. 2). Most patients suffered their injury due to a fall, predominantly the same-level fall (Table 1; Fig. 3). 71% of all patients > 65 sustained their fracture through a simple same-level fall. One in four patients < 65 years sustained their fracture in a bicycle accident. High-energy injuries were rare, causing between 6 and 11% of the all fractures depending on fracture type, with the highest proportion being the distal olecranon fractures. Men sustained high enery injuries to a higher degree than women in both patients younger and older than 65 years (Table 2). 13% of all fractures in patients < 65 were high-energy injuries. The proportion increased with increasing fracture complexity, with 20% of distal olecranon fractures being high-energy injuries in patients < 65 (Table 1).

**Table 1:** Distribution of sex, age at injury, injury mechanism and type of injury for the four fracture subgroups as well as overall fractures stratified for patients younger ≤ 65 years of age at injury and those > 65 years

	Proximal olecranon fracture (avulsion)		Central olecranon fracture (simple)		Central olecranon fracture (comminuted)		Distal olecranon fracture (unstable joint)		Overall	
	≤ 65 (*N* = 152)	> 65 (*N* = 151)	≤ 65 (*N* = 488)	> 65 (*N* = 556)	≤ 65 (*N* = 374)	> 65 (*N* = 343)	≤ 65 (*N* = 190)	> 65 (*N* = 208)	≤ 65 (*N* = 1204)	> 65 (*N* = 1258)
Sex										
Female	83 (54.6%)	102 (67.5%)	273 (55.9%)	424 (76.3%)	198 (52.9%)	265 (77.3%)	95 (50.0%)	150 (72.1%)	649 (53.9%)	941 (74.8%)
Male	69 (45.4%)	49 (32.5%)	215 (44.1%)	132 (23.7%)	176 (47.1%)	78 (22.7%)	95 (50.0%)	58 (27.9%)	555 (46.1%)	317 (25.2%)
Age at injury										
Mean (SD)	45.4 (14.3)	78.2 (8.74)	45.4 (14.5)	79.7 (8.50)	46.5 (14.0)	78.5 (8.15)	47.1 (14.3)	78.5 (8.22)	46.0 (14.3)	79.0 (8.40)
Mechanism										
Fall same level	78 (51.3%)	104 (68.9%)	244 (50.0%)	402 (72.3%)	159 (42.5%)	242 (70.6%)	86 (45.3%)	146 (70.2%)	567 (47.1%)	894 (71.1%)
Fall from height	11 (7.2%)	13 (8.6%)	39 (8.0%)	32 (5.8%)	39 (10.4%)	31 (9.0%)	22 (11.6%)	16 (7.7%)	111 (9.2%)	92 (7.3%)
Unspecified fall	12 (7.9%)	21 (13.9%)	39 (8.0%)	64 (11.5%)	25 (6.7%)	27 (7.9%)	10 (5.3%)	13 (6.2%)	86 (7.1%)	125 (9.9%)
Bicycle	26 (17.1%)	7 (4.6%)	120 (24.6%)	35 (6.3%)	104 (27.8%)	27 (7.9%)	36 (18.9%)	24 (11.5%)	286 (23.8%)	93 (7.4%)
Motorbike	2 (1.3%)	1 (0.7%)	10 (2.0%)	1 (0.2%)	10 (2.7%)	1 (0.3%)	8 (4.2%)	0 (0%)	30 (2.5%)	3 (0.2%)
Stress fracture	0 (0%)	0 (0%)	1 (0.2%)	1 (0.2%)	0 (0%)	0 (0%)	0 (0%)	2 (1.0%)	1 (0.1%)	3 (0.2%)
Other cause	23 (15.1%)	5 (3.3%)	35 (7.2%)	21 (3.8%)	37 (9.9%)	15 (4.4%)	28 (14.7%)	7 (3.4%)	123 (10.2%)	48 (3.8%)
Type										
Low energy	123 (80.9%)	140 (92.7%)	386 (79.1%)	506 (91.0%)	292 (78.1%)	303 (88.3%)	136 (71.6%)	181 (87.0%)	937 (77.8%)	1130 (89.8%)
High energy	14 (9.2%)	5 (3.3%)	50 (10.2%)	11 (2.0%)	53 (14.2%)	14 (4.1%)	38 (20.0%)	6 (2.9%)	155 (12.9%)	36 (2.9%)
Unknown	9 (5.9%)	2 (1.3%)	29 (5.9%)	15 (2.7%)	17 (4.5%)	8 (2.3%)	9 (4.7%)	5 (2.4%)	64 (5.3%)	30 (2.4%)
Not applicable	0 (0%)	0 (0%)	1 (0.2%)	1 (0.2%)	0 (0%)	0 (0%)	0 (0%)	2 (1.0%)	1 (0.1%)	3 (0.2%)
Missing	6 (3.9%)	4 (2.6%)	22 (4.5%)	23 (4.1%)	12 (3.2%)	18 (5.2%)	7 (3.7%)	14 (6.7%)	47 (3.9%)	59 (4.7%)

**Table 2:** Distribution of the type of injury for patients aged 65 years and below and those older than 65 years, further stratified for sexes

	≤ 65 years		> 65 years		Overall	
	Female (*N* = 649)	Male (*N* = 555)	Female (*N* = 941)	Male (*N* = 317)	Female (*N* = 1590)	Male (*N* = 872)
Type of injury						
High energy	67 (10.3%)	88 (15.9%)	23 (2.4%)	13 (4.1%)	90 (5.7%)	101 (11.6%)
Low energy	531 (81.8%)	406 (73.2%)	858 (91.2%)	272 (85.8%)	1389 (87.4%)	678 (77.8%)
Not applicable	1 (0.2%)	0 (0%)	2 (0.2%)	1 (0.3%)	3 (0.2%)	1 (0.1%)
Unknown	26 (4.0%)	38 (6.8%)	19 (2.0%)	11 (3.5%)	45 (2.8%)	49 (5.6%)
Missing	24 (3.7%)	23 (4.1%)	39 (4.1%)	20 (6.3%)	63 (4.0%)	43 (4.9%)

### Fracture classification

Of all fractures, 303 (12%) were proximal avulsion, 1044 (42%) simple central, 717 (29%) comminuted central and 398 (16%) distal olecranon fractures. Eighty-nine fractures (3.6%) were classified as open: 28 were Gustilo-Anderson type I, 25 type II, 15 type IIIa, 1 type IIIb, and 4 had a missing G–A classification. Men displayed an even distribution of all four fracture types in all age groups, whereas women sustained all these fractures in older age, mostly the simple or comminuted central fractures (Fig. 2).

### Treatment

Most patients suffering a simple avulsion fracture were treated nonoperatively (59%). This proportion dropped for the two central olecranon fractures (32% and 14%) and the more distal olecranon fracture (16%). The method of surgical fixation differed between groups. For the more stable proximal and simple central olecranon fractures, most patients were operated on with TBW. More than half of the operatively treated patients with more unstable fractures were treated with plate fixation (Table 3). Patients > 65 years were more often treated non-operatively for all fracture types.

**Table 3:** Treatment choice for the four fracture subgroups and overall fractures stratified for patients ≤ 65 years of age at injury and those > 65 years

	Proximal olecranon fracture (avulsion)		Central olecranon fracture (simple)		Central olecranon fracture (comminuted)		Distal olecranon fracture (unstable joint)		Overall	
	≤ 65 (*N* = 152)	> 65 (*N* = 151)	≤ 65 (*N* = 488)	> 65 (*N* = 556)	≤ 65 (*N* = 374)	> 65 (*N* = 343)	≤ 65 (*N* = 190)	> 65 (*N* = 208)	≤ 65 (*N* = 1204)	> 65 (*N* = 1258)
Treatment										
Non-operative	80 (52.6%)	98 (64.9%)	111 (22.7%)	221 (39.7%)	27 (7.2%)	70 (20.4%)	14 (7.4%)	50 (24.0%)	232 (19.3%)	439 (34.9%)
Tension band wiring	47 (30.9%)	36 (23.8%)	261 (53.5%)	250 (45.0%)	132 (35.3%)	117 (34.1%)	49 (25.8%)	64 (30.8%)	489 (40.6%)	467 (37.1%)
Screw fixation	1 (0.7%)	1 (0.7%)	2 (0.4%)	0 (0%)	2 (0.5%)	2 (0.6%)	2 (1.1%)	1 (0.5%)	7 (0.6%)	4 (0.3%)
Plate fixation	5 (3.3%)	6 (4.0%)	66 (13.5%)	40 (7.2%)	176 (47.1%)	127 (37.0%)	95 (50.0%)	67 (32.2%)	342 (28.4%)	240 (19.1%)
Combined fixation	6 (3.9%)	2 (1.3%)	13 (2.7%)	9 (1.6%)	15 (4.0%)	8 (2.3%)	9 (4.7%)	8 (3.8%)	43 (3.6%)	27 (2.1%)
Missing	13 (8.6%)	8 (5.3%)	35 (7.2%)	36 (6.5%)	22 (5.9%)	19 (5.5%)	21 (11.1%)	18 (8.7%)	91 (7.6%)	81 (6.4%)

### Seasonal variation

**Fig. 4:**
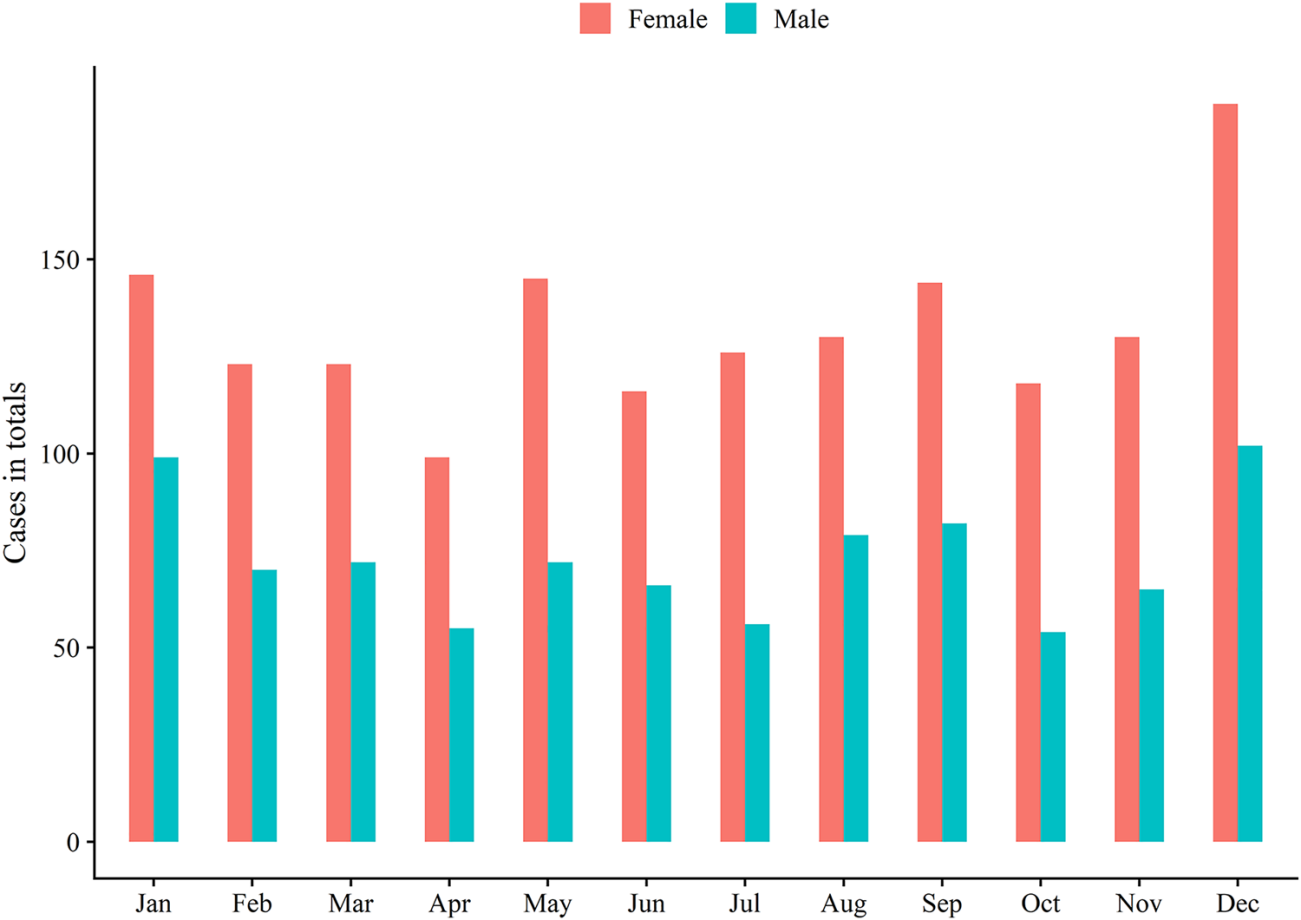
Seasonal variation of all fractures sustained in women and men

Both women and men appeared to sustain more fractures in winter (December to January) and there was an observed tendency to a slight increase in summer (May–September) (Fig. 4).

## Discussion

The main finding in this register-based descriptive study of 2462 olecranon fractures registered in the SFR between 2014 and 2018 is that most fractures occurred in elderly women due to low-energy same-level simple falls. Our study is the first to analyze the epidemiology of olecranon fractures for age and sex distribution, injury mechanisms, fracture type and treatment in a nationwide cohort. Previous investigations were restricted to single-center studies [1], with limited external validity as reference centers might treat a selected cohort of patients. In the present study of 2462 olecranon fractures, we found a larger share of women (65%) compared to the 55% reported in a previous single-center study describing 64 olecranon fractures [1]. In addition, the median age (66 years) in our study was higher.

We confirmed the common assumption that an olecranon fracture is considered a fragility fracture [1] because the leading injury mechanism is a same-level fall in an elderly woman. We found that the four assessed fracture types share roughly the same demographic patterns and injury mechanism but differ in the chosen treatment modality.

The prevalence of simple falls as the injury mechanism for olecranon fractures has been described elsewhere [1]. Transportation accidents resulting in olecranon fractures occurred mainly in patients < 65 years and most fractures were due to a bicycle accident. Bicycle commuting is a popular way of getting to work and specific bicycle routes are common. High-energy injuries were more common in men, in younger patients and in more compex fractures in our study. These young men with high-energy injuries illustrate a subgroup of patients also seen in other locations with severe injuries sustained by high-energy injury mechanisms [11, 12]. A more detailed investigation of how these accidents occur is warranted to counsel the population and decrease fracture risks.

### Fracture classification

The distribution of fractures in our study differed compared to the single-center report from Scotland, [1] where 74% of the fractures were simple central fractures and 81% central fractures. We found a larger share of comminuted central fractures (29%) compared to the study from Scotland. We also observed an almost threefold larger share of distal olecranon fractures in our material than the 6.2% in the Scottish study of 64 olecranon fractures [1].

The different settings of the studies might explain the discrepancy between the two studies: a Scandinavian context with nationwide coverage compared to an urban environment in Scotland. Moreover, the classification of fractures differed between the studies. In our study, a large number of observers (i.e. orthopedic surgeons) with a diverse range of experience from junior doctors to senior consultants performed the classification.

There are several different classification systems for olecranon fractures, e.g. Colton, Mayo, Schatzker and AO classications [13]. They all have different rationales for grouping the fractures; displacement and fracture pattern [14]; stability, displacement and comminution [15]; fracture morphology and biomechanical stability [16]; or as part of elbow fractures with information about extra, partial or complete articular fractures [17]. Further, there is newly updated version of the AO/OTA classification including more subgroups [18].

### Treatment

Non-operative treatment was done in decreasing proportion with the increasing complexity of the fracture. A larger share of patients > 65 years were treated non-operatively for all fracture types, which concurs with previous findings of good outcomes after nonoperative treatment of stable fractures in patients with low functional demands [4, 6, 19, 20]. In the more unstable fractures, TBW and plate fixation were equally commonly performed in elderly patients. In a review, implant type was not found to matter regarding risk of reoperation in elderly patients [6] and this seems not to influence the treatment choice. Plate fixation offers better biochemical properties as well as interfragmentary compression [21]. This seems to influence the choice of treatment in the younger patients with more unstable fractures, i.e. plate fixation was the preferred treatment for younger patients.

In the SFR, there is no information on type of non-operative treatment which can differ from early or direct range of motion to several weeks in cast. In line with previous recommendations, plate fixation was mainly used for unstable fractures [22]. There is no detailed information on plate type or number of plates in the SFR. This is however a study on recent fractures in the locking plate era, so the majority of plates are presumably locking plates. Recently, some centers have started to use multiple small plates in select cases, sometimes as an adjunct to locking plates. This is not a widespread practice in Sweden. However, identifying and proper handling of intermediate fracture fragments seem to be more important than the implant itself [23].

### Seasonal variation

The incidence of all fractures varied little over the year. A slightly higher occurrence of olecranon fractures in both women and men was observed in the winter, which could be linked to icy conditions during this time of year.

### Strength and limitations

Our study has obvious limitations. We used the SFR that had an 85% coverage at the end of the study period to describe the epidemiology of olecranon fractures with the by largest number of olecranon fractures described in the literature. Regrettably, we cannot identify the overall incidence of olecranon fractures given the stepwise introduction and the present completeness of the SFR in Sweden. Furthermore, the stepwise introduction effectively hindered a comparison with the base population and thus analyses on outcomes of interest.

Another limitation concerns the Mayo classification’s questionable reliability [13, 24], making comparisons difficult to establish [25]. The modified classification of the SFR allows four options and could have a favorable agreement among observers. All treating physicians and orthopedic surgeons performed the registrations and classifications in the SFR. Of note, validation studies in various segments found the classification systems to be as accurate as previous validation studies of fracture classification [7–9]. Regarding treatment details, there is no information on type of non-operative treatment, such as immobilization type or length. Neither is there information on type of and number of plates which would add to the study. However, with the current study, which was based on a nationwide register, we present relevant epidemiological data important for the surgeon (i.e. in preoperative counseling of patients) with information on general type of performed treatment. Reoperation rates in the SFR need to be verified by linking registers or by confirmation through a search in the medical files, which is outside the scope of this study.

## Conclusion

Isolated fractures of the olecranon occur mainly in elderly women after low-energy trauma. Non-operative treatment is common in simple fractures, whereas operative treatment is preferred in more complex fractures. More recently, there has been a shift towards plate fixation in the more unstable fracture patterns. Our results can help health care providers and clinicians understand isolated olecranon fractures.

## Data Availability

Data can be made available on request to the authors.

## References

[1] Duckworth AD, Clement ND, Aitken SA, Court-Brown CM, McQueen MM (2012). The epidemiology of fractures of the proximal ulna. Injury.

[2] Cabanela MEM (1993). The elbow and its disorders.

[3] Brolin TJ, Throckmorton T (2015). Olecranon fractures. Hand Clin.

[4] Duckworth AD, Bugler KE, Clement ND, Court-Brown CM, McQueen MM (2014). Nonoperative management of displaced olecranon fractures in low-demand elderly patients. J Bone Joint Surg Am.

[5] García-Elvira R, Vives-Barquiel MA, Camacho-Carrasco P, Ballesteros-Betancourt JR, García-Tarriño R, Domingo-Trepat A (2020). Olecranon mayo IIA fractures treated with transosseous high strength suture: a series of 29 cases. Injury.

[6] Chen MJ, Campbell ST, Finlay AK, Duckworth AD, Bishop JA, Gardner MJ (2021). Surgical and nonoperative management of olecranon fractures in the elderly: a systematic review and meta-analysis. J Orthop Trauma.

[7] Juto H, Moller M, Wennergren D, Edin K, Apelqvist I, Morberg P (2016). Substantial accuracy of fracture classification in the Swedish Fracture Register: evaluation of AO/OTA-classification in 152 ankle fractures. Injury.

[8] Wennergren D, Ekholm C, Sundfeldt M, Karlsson J, Bhandari M, Moller M (2016). High reliability in classification of tibia fractures in the Swedish Fracture Register. Injury.

[9] Wennergren D, Stjernstrom S, Moller M, Sundfeldt M, Ekholm C (2017). Validity of humerus fracture classification in the Swedish fracture register. BMC Musculoskelet Disord.

[10] R Core Team. R: a language and environment for statistical computing. http://www.R-project.org/. Accessed 9 Dec 2019

[11] Wennergren D, Bergdahl C, Ekelund J, Juto H, Sundfeldt M, Moller M (2018). Epidemiology and incidence of tibia fractures in the Swedish Fracture Register. Injury.

[12] Rydberg EM, Zorko T, Sundfeldt M, Moller M, Wennergren D (2020). Classification and treatment of lateral malleolar fractures—a single-center analysis of 439 ankle fractures using the Swedish Fracture Register. BMC Musculoskelet Disord.

[13] Benetton CA, Cesa G, El-Kouba Junior G, Ferreira AP, Vissoci JR, Pietrobon R (2015). Agreement of olecranon fractures before and after the exposure to four classification systems. J Shoulder Elbow Surg.

[14] Colton CL (1973). Fractures of the olecranon in adults: classification and management. Injury.

[15] Morrey BF (1995). Current concepts in the treatment of fractures of the radial head, the olecranon, and the coronoid. Instr Course Lect.

[16] Schatzker J (2005). Fractures of the olecranon. The rationale of operative fracrture care.

[17] Mueller ME, Allgower M, Schneider R (1991). Manual of internal fixation: techniques recommended by the AO-AFIF group.

[18] Meinberg EG, Agel J, Roberts CS, Karam MD, Kellam JF (2018). Fracture and dislocation classification compendium-2018. J Orthop Trauma.

[19] Duckworth AD, Clement ND, McEachan JE, White TO, Court-Brown CM, McQueen MM (2017). Prospective randomised trial of non-operative versus operative management of olecranon fractures in the elderly. Bone Joint J.

[20] Marot V, Bayle-Iniguez X, Cavaignac E, Bonnevialle N, Mansat P, Murgier J (2018). Results of non-operative treatment of olecranon fracture in over 75-year-olds. Orthop Traumatol Surg Res.

[21] Wilson J, Bajwa A, Kamath V, Rangan A (2011). Biomechanical comparison of interfragmentary compression in transverse fractures of the olecranon. J Bone Joint Surg Br.

[22] Powell AJ, Farhan-Alanie OM, Bryceland JK, Nunn T (2017). The treatment of olecranon fractures in adults. Musculoskelet Surg.

[23] von Rüden C, Woltmann A, Hierholzer C, Trentz O, Bühren V (2011). The pivotal role of the intermediate fragment in initial operative treatment of olecranon fractures. J Orthop Surg Res.

[24] Schliemann B, Raschke MJ, Groene P, Weimann A, Wahnert D, Lenschow S (2014). Comparison of tension band wiring and precontoured locking compression plate fixation in Mayo type IIA olecranon fractures. Acta Orthop Belg.

[25] Sullivan CW, Desai K (2019). Classifications in brief: Mayo classification of olecranon fractures. Clin Orthop Relat Res.

